# Perceived Relative Harm of Selected Cigarettes and Non-Cigarette Tobacco Products—A Study of Young People from a Socio-Economically Disadvantaged Rural Area in Poland

**DOI:** 10.3390/ijerph13090885

**Published:** 2016-09-06

**Authors:** Dorota Kaleta, Kinga Polanska, Leokadia Bak-Romaniszyn, Piotr Wojtysiak

**Affiliations:** 1 Department of Tobacco Control, Preventive Medicine Department, Medical University of Lodz, Lodz 90-752, Poland; kinga.polanska@umed.lodz.pl (K.P.); pwojtysiak@wp.pl (P.W.); 2 Department of Nutrition in Digestive Tract Diseases, Medical University of Lodz, Lodz 93-338, Poland; leokadia.bak-romaniszyn@umed.lodz.pl

**Keywords:** tobacco control, harm reduction, public health education, health communication, cigarettes, electronic cigarettes, smokeless tobacco, water pipe

## Abstract

The perceived health risk of recently introduced nicotine and tobacco products may influence both their uptake and continued use. The aim of the study was to assess how adolescents rate relative harmfulness of slim and menthol cigarettes, water pipes, e-cigarettes, and smokeless tobacco compared to regular cigarettes. Cross-sectional survey data from students aged 13–19 years from Piotrkowski district, Poland were analyzed. Among the sample of 4050 students, 3552 respondents completed anonymous, confidential, self-administered questionnaire adapted from the Global Youth Tobacco Survey (GYTS). The study results indicate that the students perceived slim cigarettes and menthol cigarettes as less harmful, which is in line with the message created by tobacco companies. On the other hand, less popular products such as water pipes and smokeless tobacco were considered as more harmful. The current study indicates insufficient and misleading perception of harmfulness of different tobacco/nicotine products available on the Polish market. Simultaneously, there is insufficient countrywide public health education in this matter. Preventive measures are necessary to discourage young people from smoking uptake and to ensure that potential consumers can, based on objective data, make informed decisions about cigarettes and non-cigarette tobacco products.

## 1. Introduction

Smoking kills more Europeans than any other avoidable factor. Compared to the rest of the world, the European Region has the highest rate of smoking and the highest proportion of deaths attributable to tobacco. On average, 32% of adults smoke, and 16% of all deaths in adults aged over 30 in the WHO European Region are due to tobacco use [1].

In Poland, adult smoking rates continue to decrease slightly, but in 2010, 33.5% of adult men (5.2 million) and 21.0% of adult women (3.5 million) continued to smoke [2,3,4]. Conversely, in recent years, the prevalence of smoking tobacco in adolescents in Poland increased significantly (from 23.9% in 2010–2011 to 38.0% in 2013–2014) [5]. Moreover, the prevalence of e-cigarettes use among teenagers increased from 5.5% in 2010 to 29.9% in 2014—similar to the rate of using both tobacco and e-cigarettes (3.6% to 21.8%, respectively).

The youth initiation and continuous use of cigarettes as well as non-cigarette nicotine or tobacco products remain a subject of public health concern with special attention to vulnerable groups. Reducing smoking in more disadvantaged areas is a particular challenge as people are more likely to be exposed to smoking at home, more likely to take up smoking at a younger age and to become smokers themselves [6,7]. Tobacco control impact on this priority group is undermined by tobacco marketing strategies, which are specifically targeted at young people.

On the Polish market there are many regulated tobacco products including: different types of cigarettes, cigars, cigarillos, water pipes, and smokeless tobacco [8,9]. Electronic nicotine delivery systems (e-cigarettes) are also rapidly gaining popularity. Tobacco/nicotine products are elaborately designed to initiate smoking and to weaken efforts on quitting smoking. The tobacco industry offered, e.g., so called “low tar” and “light” cigarettes and thus, created a false image that those cigarettes are “healthier” or “less addictive”. Following the ban on the use of such descriptions in Poland, tobacco manufacturers have used alternative brand imagery to mislead consumers [10]. Different brands, such as “slim” or “flavored” cigarettes, are introduced to attract new and maintain existing consumers. Menthol, candy, alcohol, and chocolate flavors make these products attractive to teenagers and young women. 

According to the existing knowledge, all tobacco products—smokeless, smoked tobacco, and cigarette substitutes—are harmful and addictive and all can cause disease and death [11]. However, current scientific evidence also indicates that tobacco products differ in related health harm [12,13,14]. Combustible tobacco products (including all types of cigarettes without any exception) inhaled into the lungs are ranked as the most harmful, while non-combustible products are assessed to be relatively less harmful [15]. In this respect, slim and menthol cigarettes can be considered as harmful as traditional cigarettes. There is general agreement in scientific community that the health hazards from smokeless tobacco products are lower than those of cigarette smoking. Similarly, a growing number of studies suggest higher safety of e-cigarettes compared to traditional cigarettes use and are promoted as the effective option of harm-reducing strategies [16]. The literature on the health effects of water pipe use is less robust than those on cigarettes or smokeless tobacco, but indicates that intake of smoke are orders of magnitude higher than of cigarettes with equivalent nicotine exposure [12,13,14,15].

Knowledge and individual perceptions about adverse effects of all forms of tobacco exert direct influence on the level of tobacco consumption and potential for implementing harm reduction policies in various socio-demographic groups [17]. Community-centered approaches are some of the available methods that can be used to improve health and well-being. However, at the same time, remote rural-dwellings compared to urban centers in Poland are usually less covered by educational and promotional activities as well as tobacco control measures.

Previous research has revealed misperceptions of the relative harmfulness of selected brands of cigarettes and cigarette substitutes in different populations [18,19,20,21]. In Poland, little is known about this issue, especially in relation to the assessment on how socio-economically disadvantaged rural adolescent sub-population perceive the risks of using various available tobacco products.

The aim of the study was to assess how adolescents from a rural area of Poland rate relative harmfulness of smoked tobacco products—selected types of cigarettes (including slim and menthol), water pipes, smokeless tobacco (tabaka) and e-cigarettes—compared to traditional cigarettes.

## 2. Materials and Methods

During the period from November 2014 to May 2015, a cross-sectional study was conducted among secondary and high school students (aged 13–19) from Piotrkowski district, which is a socially disadvantaged rural area in central Poland (Lodzkie voivodeship—administrative region of central Poland). According to the state as of the year 2013, there were 91,618 residents, including 45,223 men and 46,395 women, living on the premises of the district with more than 90% of the residents representing rural area. The analysis performed by the United Nations Development Program (UNDP) defined Piotrkowski district as the 11th position of all 314 rural districts with the lowest indicators of social development in Poland. Local Human Development Index (LHDI)—covering three indicators: Health Index, Education Index, and Welfare Index—was 25.97, with Health Index (HI) = 26.50, whereas the discussed indicators for Lodzkie voivodeship were 39.28 and 31.48 respectively [22].

Before the study was implemented, approvals from the Department of Education of Piotrkowski district and from directors of educational institutions where the research was carried out, were obtained (Project Identification Code: RNN/730/14/KB). All of the 16 secondary and 5 high schools in Piotrkowski district were invited to participate in the project, and agreed to be involved in the study. Data collection was supervised and coordinated by the principal investigator and 10 field supervisors.

In total, 4050 students were invited to participate. Among this group, the filled-in questionnaires were returned by 3552 respondents (88%).

The study obtained a positive opinion from the Bioethics Committee of Medical University in Lodz. A written informed consent was obtained from all the participants and their parents or legal guardians.

An anonymous, self-administered questionnaire, filled in by the students during regular classes, consisted 84 questions, including core questions from original Global Youth Tobacco Survey (GYTS) and additional country-specific questions. The questionnaire covered socio-demographic data (age, gender, parental education), information about cigarette use (including tobacco and e-cigarettes), environmental tobacco smoke exposure, and knowledge and attitudes towards cigarette use as well as existing legislation and prevention activities in this field.

Adolescents’ smoking behavior was assessed via the question: “Have you ever smoked cigarettes?” Those who responded “no” were considered as never cigarette smokers. Ever cigarette smokers were defined as those who have tried cigarettes but did not smoke them within the month preceding the study. These two categories (never and ever smokers) were grouped in the analysis as non-smokers. Students who smoked cigarettes at least once during the past 30 days were classified as current smokers. Similar procedures were used to categorize e-cigarette users.

The youths’ perception of selected brands of traditional cigarettes and alternative nicotine products were assessed based on the items adapted from Smith et al. 2007 [23]. Adolescents were asked (separately for each product): “Compared with traditional cigarettes how harmful do you think menthol, slim cigarettes, smokeless tobacco, water pipe, and e-cigarettes are?” with the possible answers: much less harmful, less harmful, just as harmful, more harmful, or much more harmful. In the analysis, the answers were converted into the points (from 1 = much less harmful to 5 = much more harmful). Finally, the points were used as continuous variables to estimate mean rating for each product. Taking into account that cigarettes and e-cigarettes are the most common products among adolescents in Poland, the analyses were performed for the smokers (those who indicated cigarette smoking but not e-cigarette use), e-cigarette users (those who used e-cigarettes but did not smoke traditional cigarettes), and dual users (those who smoked traditional cigarettes and used e-cigarettes) vs. non-smokers (neither smokers of traditional cigarettes nor e-cigarette users).

The data were entered into the Excel data analysis software on a daily basis by field investigators and then submitted to a supervisor. Once the data collection process were completed, 5% of the records were randomly checked to confirm that they were clearly recorded, complete, and consistent across responses. Data set is provided in supplementary materials (Supplementary Material 1: Sheet S1 and S2). The results are presented as overall mean scores (±SD) on harmfulness across various products and scores presented for gender and smoker/e-cigarette status. Significance of differences between subgroups was tested using the independent sample *t* tests, for gender comparison, and Kruskal-Wallis ANOVA (providing robustness against subgroup heterogeneity) and Dunett test (for multiple comparisons), for smoking/e-cigarette status. Finally, to assess the perceived harmfulness of the products, the linear regression model was applied.

## 3. Results

Descriptive data for the perceived harmfulness of tobacco/nicotine products are presented in supplementary materials (Supplementary Material 2: Table S1). Table 1 and Table 2 present the mean scores for the perceived harmfulness of the five types of analyzed tobacco/nicotine products compared to traditional cigarettes (with 3 indicating that the products are perceived as harmful as traditional cigarettes). Smokeless tobacco was indicated as the most harmful form of nicotine product among those included, with a mean score of 2.84 (±1.11) from the five-point scale (Table 1). At the lower end the adolescents perceived slim cigarettes (mean 1.96 ± 1.03). Boys and girls scored the harmfulness of menthol cigarettes, smokeless tobacco and water pipe compared to traditional cigarettes differently (*p* ≤ 0.001). Perception of tobacco/nicotine products compared to traditional cigarettes was also different depending on the smoker/e-cigarette status (Table 2). As an example, the smokers of traditional cigarettes indicated significantly lower mean score for perceived harmfulness of smokeless tobacco (*p* = 0.04) and higher for e-cigarettes (*p* < 0.001) than the e-cigarette users did.

In the multivariate linear regression model, higher age predicted lower levels of perceived harmfulness in smokeless tobacco and water pipe, and higher levels in e-cigarettes (Table 3) compared to traditional cigarettes (*p* ≤ 0.001). The boys rated harmfulness higher on menthol cigarettes and smokeless tobacco and lower on water pipe compared to traditional cigarettes comparing to the girls (*p* < 0.001). The smokers of traditional cigarettes reported more harmfulness (compared to traditional cigarettes) in menthol, slim cigarettes, and e-cigarettes comparing to the non-smokers (*p* ≤ 0.03). The e-cigarette users compared to the non-users gave significantly more points for slim cigarettes and smokeless tobacco and fewer for water pipe as their perception of harmfulness of these products compared to traditional cigarettes (*p* ≤ 0.008). A similar pattern was observed in the dual users’ perception of harmfulness of tobacco/nicotine products. Analysis stratified by gender is presented in supplementary materials (Supplementary Material 2: Table S2).

## 4. Discussion

According to the Surgeon General conclusion in 2014, the greatest danger to public health is from cigarettes and other combustible products [14,15]. At the same time, smokeless tobacco and alternative nicotine delivery systems have improved and it is evidence-based that they are less harmful than cigarettes [14]. The current study, similar to other studies in this field, pointed out that adolescents do not have an expert understanding of harmfulness of different tobacco/nicotine products [12,14,18,24,25,26,27,28,29,30,31,32,33,34,35]. Our results indicate that students perceived slim cigarettes and menthol cigarettes as less harmful. On the other hand, the less known and popular products such as water pipe and smokeless tobacco were considered more harmful. These results reflect attractiveness of the products and intensity of the pro- and anti-tobacco activities. The current study also underlines differences in perception of harmfulness of different types of nicotine products by age, gender, and cigarette/e-cigarette use, which allows distinction of vulnerable groups for education and intervention activities.

The study results demonstrate that youths’ perception of harmfulness of different tobacco/nicotine products does not reflect scientific evidence and thus perceptions might be reflective of tobacco industry marketing. Tobacco companies are obligated by law to inform the substance content and harmfulness of their products but only to some extent [8]. In addition, e-cigarettes are not covered by Polish legislation, and are easily accessible to the minors without any restrictions. This creates the situation in which society receives inconsistent information mostly based on crude, unjustified claims of the product risk [36,37]. Tobacco companies give numerous reasons for failing to disclose the truth about their products fully [11]. Although some cigarette manufacturers reveal some details regarding substances that can be found in their products, they do not list a large number of ingredients in the final cigarette including herbicides, pesticides, fertilizer, arsenic, heavy metals, and many other substances that are in the glue, paper, or filters [38]. According to the MANKO NGOs report analyzing tobacco industry strategies in Poland, one may notice the diversity of tactics in use [37]. With the awareness of how efficient advertising is as a means of manipulation, the tobacco sector has worked out a range of strategies enabling it to legally promote tobacco companies and their products. Because of the restrictions on the tobacco product market, the undertaken steps are creative and meticulously planned. Direct and indirect initiatives in the field of public relations include participating in high-profile events such as large-scale events, but also internal initiatives addressed to the in-house staff. Strategies of tobacco companies operating in Poland are consistent with international standards. They also take into account domestic norms as well as local characteristics of the Polish market. The most common practices represented by tobacco industry are: creating a positive image, which allows concluding cooperation with various media (including social media), as well as direct and indirect advertising. Brand stretching, product advertising, supporting public events, trade fairs, recruitment strategies and hiring, awards, and rankings are common activities observed in Poland. These practices can influence young people’s knowledge, attitudes, and perception of tobacco.

The existing public health campaigns concentrate their efforts on health risk relative to traditional cigarettes and, in this context, most emphasis is put on the risk of lung cancer and cardiovascular diseases, whereas other health consequences are less frequently pointed out [17]. It also needs to be emphasized that even though smokeless tobacco does not generate dangerous products of combustion, they anyhow contain addictive levels of nicotine, many carcinogens, heavy metals, and other toxins [11]. Several companies have aggressively marketed them to cigarette smokers as an alternative in situations in which smoking is not allowed, and thus, promoting the dual use of smokeless and smoked products. On the other hand, smokeless tobacco may cause harm as a gateway to smoking [24]. Taking into consideration popularity of some products such as e-cigarettes, the public health message, especially that addressed to young people, should not only focus on their health risk in relation to traditional cigarettes, but it should also be pointed out that these products, as products containing nicotine, can cause addiction.

The current study indicates differences in the perception of harmfulness of different types of nicotine products by age, gender, and cigarette/e-cigarette use. It needs to be underlined that in the studied population we observed high percentages of young people who declared current (within 30 days) cigarette smoking (29.4%) and e-cigarette use (27.4%). Such results are in line with the recent studies conducted in Poland [5,39,40]. What is interesting is that the e-cigarette users in our study perceived the products that they use as less harmful and smokeless tobacco as more harmful in relation to traditional cigarettes when compared to the smokers of traditional cigarettes. It is not possible to judge if such an opinion motivates the students to use or not to use such products. It is not under discussion that e-cigarettes are safer compared to traditional cigarettes but for adolescents, the use of nicotine-containing e-liquids creates the risk of developing dependence and initiation of tobacco use. The other concern is related to the dual use (e-cigarettes and conventional cigarettes), in which individuals are exposed to, and addicted to higher levels of nicotine (in the current study, it is represented by 58% of the population). Consequently, as they believe that by using e-cigarettes they reduce their conventional tobacco use and are getting healthier, their chances of cessation are reduced and cessation attempts are delayed [41].

Being a smoker was associated with a higher rating of harm for slim, menthol, and e-cigarettes, which also indicates the need for further education in this field. In addition, with the increasing age the perception of harmfulness of the products was not consistently changing. This can reflect students’ maturity and ability to perceive the risk presented to the public. Males and females in our study scaled the studied products differently but not in a similar direction, whereas Norwegian boys reported lower harmfulness for all the products when compared to girls [24]. This can reflect socio-cultural differences between the countries and it can also indicate to what extent young people are influenced and susceptible to tobacco marketing. Moreover, objective, comprehensive information about harmfulness of selected brands is rarely disseminated in Poland.

The data used in the current analysis are based on a large number of respondents from the entire area of Piotrkowski district, assuring generalizability of the results for rural areas, especially those who are socially disadvantaged. In addition, this study showed the situation in small rural communities usually poorly covered by surveillance. However, its applicability to urban areas (or other regions) may be limited and it cannot be assumed that all the youth share the same views about the relative harmfulness of different tobacco/nicotine products. The study protocol and questionnaire are based on valid tools and GYTS standards developed by experts in the field, which enables comparison between countries and trend assessments. The study has some limitations that need to be pointed. All estimates in our assessment were based on self-reports, which might be affected by reporting bias (this is mostly related to tobacco smoking and e-cigarette use). The students may also under- or over-report their assessment of harmfulness of selected tobacco products. The assessment of the harmfulness of different tobacco/nicotine products in relation to traditional cigarettes (not the rating of each product) can also create some limitations of the study. It is likely that non-smokers think that cigarettes are more harmful than smokers which may mean that their scale from 1 to 5 is different from a smoker’s scale, e.g., non-smokers might think that menthol cigarettes are less harmful but, overall, they might think they are more harmful because they may assess any cigarette as very harmful to health.

## 5. Conclusions

The study indicates that the youth are not well informed about the relative harmfulness of different tobacco/nicotine products. It clearly indicates that preventive measures are needed to discourage smoking uptake by young people and to ensure that potential consumers can, based on objective data, take informed decisions about cigarette and non-cigarette tobacco products. Dissemination of reliable health information is one of the key tools of public health action, and it is generally assumed that informing the public about health-compromising behavior will help to prevent such behavior over time. Adolescents seem to be a priority group, and targeting initiation at this group is particularly important, as the majority of adult smokers started smoking in their youth. The local government and schools should play a major role in building skills and knowledge needed to improve health and well-being among students. Further research is needed to provide more in-depth evidence on this topic in Poland.

## Figures and Tables

**Table 1:** Mean scores for the perceived harmfulness of the selected tobacco/nicotine products by gender.

Product *	Overall	Males	Females	t (*p* Value)
	Mean ± SD			
Menthol cigarettes	2.23 ± 1.00	2.31 ± 1.07	2.12 ± 0.90	5.69 (<0.001)
Slim cigarettes	1.96 ± 1.03	1.95 ± 0.99	1.97 ± 1.08	−0.61 (0.54)
Smokeless tobacco	2.84 ± 1.11	2.91 ± 1.18	2.75 ± 1.00	4.36 (<0.001)
Water pipe	2.54 ± 1.12	2.40 ± 1.07	2.71 ± 1.14	−8.39 (<0.001)
E-cigarettes	2.34 ± 1.02	2.34 ± 1.01	2.34 ± 1.03	0.18 (0.86)

* scoring has been given to each product based on their harmfulness compared to traditional cigarettes with 1 = much less harmful; 2 = less harmful; 3 = as harmful; 4 = more harmful; 5 = much more harmful (the points were used as continuous variables to estimate mean rating for each product). SD, standard deviation.

**Table 2:** Mean scores for the perceived harmfulness of the selected tobacco/nicotine products by a participant’s traditional cigarette/e-cigarette use status.

Product *	Smoker (a)	E-Cigarettes User (b)	Dual User (c)	Non-Smoker (d)	H (*p* Value) ^#^	Z (*p* Value) ^^^
	Mean ± SD					
Menthol cigarettes	2.33 ± 1.00	2.27 ± 1.06	2.18 ± 1.11	2.22 ± 0.96	11.86 (0.008)	(a)-(c): 3.20 (0.008)
Slim cigarettes	2.10 ± 1.20	2.02 ± 1.07	2.14 ± 1.08	1.86 ± 0.95	37.99 (<0.001)	(a)-(d): 3.01 (0.02) (c)-(d): 5.36 (<0.001)
Smokeless tobacco	2.86 ± 0.83	3.09 ± 1.31	2.89 ± 1.27	2.78 ± 1.07	27.13 (<0.001)	(a)-(b): 2.68 (0.04) (b)-(d): 4.77 (<0.001)
Water pipe	2.52 ± 1.01	2.43 ± 1.04	2.32 ± 1.12	2.62 ± 1.14	48.33 (<0.001)	(a)-(c): 3.24 (0.007) (b)-(d): 3.22 (0.008) (c)-(d): 6.26 (<0.001)
E-cigarettes	2.70 ± 1.17	2.27 ± 0.78	2.31 ± 0.98	2.28 ± 1.02	45.16 (<0.001)	(a)-(b): 4.29 (<0.001) (a)-(c): 4.89 (<0.001) (a)-(d): 6.34 (<0.001)

* scoring has been given to each product based on their harmfulness compared to traditional cigarettes with 1 = much less harmful; 2 = less harmful; 3 = as harmful; 4 = more harmful; 5 = much more harmful (the points were used as continuous variables to estimate mean rating for each product). SD, standard deviation. (a) The youth who indicated traditional cigarette smoking but not e-cigarette use (*n* = 443); (b) the youth who used e-cigarettes but did not smoke traditional cigarettes (*n* = 374); (c) the youth who smoked traditional cigarettes and used e-cigarettes (*n* = 601); (d) neither smokers of traditional cigarettes nor e-cigarette users (*n* = 2134). ^#^ H-statistics in ANOVA Kruskal-Wallis test; ^^^ z-statistics in Dunett test.

**Table 3:** Associations between the perceived harmfulness of tobacco/nicotine products and age, gender, smoking, and e-cigarette use ^#^.

Product *	Age	Gender (Male vs. Female)	Smoker (a) vs. Non-Smoker (d)	E-Cigarettes User (b) vs. Non-Smoker (d)	Dual User (c) vs. Non-Smoker (d)
	β (*p* Value)				
Menthol cigarettes	0.008 (0.64)	0.098 (<0.001)	0.038 (0.03)	0.010 (0.55)	−0.025 (0.15)
Slim cigarettes	−0.012 (0.49)	−0.022 (0.21)	0.079 (<0.001)	0.048 (0.005)	0.105 (<0.001)
Smokeless tobacco	−0.14 (<0.001)	0.068 (<0.001)	0.033 (0.05)	0.080 (<0.001)	0.034 (0.047)
Water pipe	−0.13 (<0.001)	−0.13 (<0.001)	−0.019 (0.26)	−0.045 (0.008)	−0.081 (<0.001)
E-cigarettes	0.077 (<0.001)	0.004 (0.79)	0.13 (<0.001)	−0.003 (0.88)	0.006 (0.72)

* scoring has been given to each product based on their harmfulness compared to traditional cigarettes with 1 = much less harmful; 2 = less harmful; 3 = as harmful; 4 = more harmful; 5 = much more harmful (the points were used as continuous variables to estimate mean rating for each product). (a) the youth who indicated traditional cigarette smoking but not e-cigarette use (*n* = 443); (b) the youth who used e-cigarettes but did not smoke traditional cigarettes (*n* = 374); (c) the youth who smoked traditional cigarettes and used e-cigarettes (*n* = 601); (d) neither smokers of traditional cigarettes nor e-cigarette users (*n* = 2134). ^#^ regression coefficients (β) presented for each variable reflect their association with perceived harmfulness after adjustment for confounding from the other factors.

## Supplementary Materials

The following are available online at www.mdpi.com/1660-4601/13/9/885/s1, Supplementary Material 1: Sheet S1: data set for high school students, Sheet S2: data set for secondary school students; Supplementary Material 2: Table S1: Perceived harmfulness of tobacco/nicotine products, Table S2: Mean scores for the perceived harmfulness of the selected tobacco/nicotine products by a participant’s cigarette/e-cigarette use status stratified by gender.
