# Supplementary material for: Perceived Relative Harm of Selected Cigarettes and Non-Cigarette Tobacco Products—A Study of Young People from a Socio-Economically Disadvantaged Rural Area in Poland
Source: Int J Environ Res Public Health. 2016 Sep 6;13(9):885. doi: 10.3390/ijerph13090885 (PMC5036718; doi:10.3390/ijerph13090885)
Supplement: Supplementary file 2 [file ijerph-13-00885-s002.pdf]

Supplementary Materials: The Youth's Perceived Relative Harm of Selected Cigarettes and Non-Cigarette Tobacco Products—A Study from A Socio-Economically Disadvantaged Rural Area in Poland

Dorota Kaleta, Kinga Polanska, Leokadia Bak-Romaniszyn and Piotr Wojtysiak

Table S1. Perceived harmfulness of tobacco/nicotine products.

Harmfulness of Selected Products		Menthol Cigarettes			Slim Cigarettes			Smokeless Tobacco			Water Pipe			E-Cigarettes		
		Less Harmful	As Harmful	More Harmful	Less Harmful	As Harmful	More Harmful	Less Harmful	As Harmful	More Harmful	Less Harmful	As Harmful	More Harmful	Less Harmful	As Harmful	More Harmful
Overall *N* = 3552	*n*	1934	1430	188	2623	701	228	1160	1570	822	1563	1482	507	1875	1387	290
	%	54.4	40.3	5.3	73.8	19.7	6.4	32.7	44.2	23.1	44.0	41.7	14.3	52.8	39.0	8.2
	95%CI	52.8–56.1	38.7–41.9	4.6–6.1	72.4–75.3	18.4–21.0	5.6–7.3	31.1–34.2	42.6–45.8	21.8–24.6	42.4–45.7	40.1–43.3	13.2–15.5	51.1–54.4	37.4–40.6	7.3–9.1
Smoker (a) *N* = 443	n	216	201	26	301	101	41	119	248	76	221	150	72	182	184	77
	%	48.7	45.4	5.9	67.9	22.8	9.3	26.9	56.0	17.1	49.9	33.9	16.2	41.1	41.5	17.4
	95%CI	44.0–53.5	40.8–50.0	3.9–8.6	63.4–72.2	18.9–26.7	6.8–12.4	22.8–31.3	51.4–60.6	13.8–21.1	45.1–54.6	29.5–38.3	13.0–20.1	36.5–45.8	36.9–46.1	14.0–21.3
E–Cigarettes User (b) *N* = 374	n	205	142	27	259	80	35	106	128	140	191	145	38	198	176	0
	%	54.8	38.0	7.2	69.3	21.4	9.3	28.3	34.2	37.4	51.1	38.8	10.2	52.9	47.1	0
	95%CI	49.6–59.9	33.1–42.9	4.9–10.5	64.3–73.8	17.2–25.6	6.7–12.9	23.9–33.3	23.4–39.0	32.6–42.6	45.9–56.2	33.9–43.7	7.4–13.8	47.8–58.1	42.0–52.2	0
Dual User (c) *N* = 601	n	398	135	68	393	146	62	209	210	182	345	184	72	329	229	43
	%	66.2	22.5	11.3	65.4	24.3	10.3	34.8	34.9	30.3	57.4	30.6	12.0	54.7	38.1	7.2
	95%CI	62.3–70.0	19.2–25.8	8.9–14.2	61.4–69.2	20.9–27.7	8.1–13.1	31.0–38.8	31.1–38.7	26.7–34.2	53.3–61.4	26.9–34.3	9.5–14.9	50.7–58.8	34.2–42.0	5.3–9.6
Non-smoker (d) *N* = 2134	n	1115	952	67	1670	374	90	726	984	424	806	1003	325	1166	798	170
	%	52.2	44.6	3.1	78.3	17.5	4.2	34.0	46.1	19.9	37.8	47.0	15.2	54.6	37.4	8.0
	95%CI	50.1–54.4	42.5–46.7	2.5–4.0	76.4–80.0	15.9–19.1	3.4–5.2	32.0–36.1	44.0–48.2	18.2–21.6	35.7–39.9	44.9–49.1	13.8–16.8	52.5–56.8	35.4–39.5	6.9–9.2

(a) the youth who indicated traditional cigarette smoking but not e-cigarette use; (b) the youth who used e-cigarettes but did not smoke traditional cigarettes; (c) the youth who smoked traditional cigarettes and used e-cigarettes; (d) neither smokers of traditional cigarettes nor e-cigarette users.

Table S2. Mean scores for the perceived harmfulness of the selected tobacco/nicotine products by a participant's cigarette/e-cigarette use status stratified by gender.

Product *	Smoker (a)	E-Cigarettes User (b)	Dual User (c)	Non-Smoker (d)	H (*p* Value) #	Z (*p* Value) ^
	Mean ± SD					
Menthol cigarettes						
Men	2.34 ± 1.07	2.34 ± 1.19	2.22 ± 1.22	2.33 ± 0.98	10.69 (0.01)	(c)-(d:) 3.0 (0.01)
Women	2.32 ± 0.93	2.14 ± 0.78	2.08 ± 0.83	2.08 ± 0.92	14.19 (0.003)	(a)-(c): 2.82 (0.03)<br>(a)-(d): 3.44 (0.004)
Slim cigarettes						
Men	2.00 ± 1.08	1.91 ± 1.01	2.04 ± 1.01	1.91 ± 0.96	6.17 (0.10)	
Women	2.22 ± 1.32	2.1 ± 1.15	2.36 ± 1.20	1.81 ± 0.95	52.26 (<0.001)	(a)-(d): 3.46 (0.003)<br>(b)-(d): 3.66 (0.002)<br>(c)-(d): 5.73 ($p < 0.001$)
Smokeless tobacco						
Men	2.94 ± 0.88	3.14 ± 1.38	2.88 ± 1.38	2.87 ± 1.11	11.50 (0.009)	(b)-(c): 2.63 (0.049)<br>(b)-(d): 3.19 (0.009)
Women	2.78 ± 0.76	3.01 ± 1.17	2.90 ± 0.99	2.68 ± 1.02	23.26 (<0.001)	(b)-(d): 3.49 (0.003)<br>(c)-(d): 3.23 (0.0075)
Water pipe						
Men	2.48 ± 1.00	2.50 ± 1.03	2.26 ± 1.12	2.41 ± 1.07	14.91 (0.002)	(a)-(c): 2.89 (0.02)<br>(b)-(c): 2.86 (0.03)<br>(c)-(d): 3.13 (0.01)
Women	2.57 ± 1.03	2.32 ± 1.03	2.44 ± 1.11	2.85 ± 1.16	51.99 (<0.001)	(a)-(d): 3.21 (0.008)<br>(b)-(d): 5.18 (<0.001)<br>(c)-(d): 4.66 ($p < 0.001$)
e–cigarettes						
Men	2.80 ± 1.05	2.24 ± 0.80	2.24 ± 1.02	2.30 ± 1.02	50.818 (<0.001)	(a)-(b): 5.05 ($p < 0.001$)<br>(a)-(c): 6.27 ($p < 0.001$)<br>(a)-(d): 6.15 ($p < 0.001$)
Women	2.58 ± 1.28	2.32 ± 0.73	2.46 ± 0.91	2.26 ± 1.02	13.82 (0.0032)	(a)-(d): 2.66 (0.05)<br>(c)-(d): 2.68 (0.04)

* scoring has been given to each product based on their harmfulness compared to traditional cigarettes with 1 = much less harmful; 2 = less harmful; 3 = as harmful; 4 = more harmful; 5 = much more harmful (the points were used as continuous variables to estimate mean rating for each product); SD, standard deviation. (a) the youth who indicated traditional cigarette smoking but not e-cigarette use (n = 443); (b) the youth who used e-cigarettes but did not smoke traditional cigarettes (n = 374); (c) the youth who smoked traditional cigarettes and used e-cigarettes (n = 601); (d) neither smokers of traditional cigarettes nor e-cigarette users (n = 2134). # H-statistics in ANOVA Kruskal-Wallis test; ^ z-statistics in Dunett test.

© 2016 by the authors; licensee MDPI, Basel, Switzerland. This article is an open access article distributed under the terms and conditions of the Creative Commons by Attribution (CC-BY) license (http://creativecommons.org/licenses/by/4.0/).
